# Molecular, Physiological and Hematological Responses of Crossbred Dairy Cattle in a Tropical Savanna Climate

**DOI:** 10.3390/biology12010026

**Published:** 2022-12-23

**Authors:** Silpa Mullakkalparambil Velayudhan, Kerstin Brügemann, Shahin Alam, Tong Yin, Chinnasamy Devaraj, Veerasamy Sejian, Eva Schlecht, Sven König

**Affiliations:** 1 Institute of Animal Breeding and Genetics, Justus-Liebig-University Gießen, Ludwigstraße 21 b, 35390 Gießen, Germany; 2 Animal Husbandry in the Tropics and Subtropics, University of Kassel and Georg-August-Universität Göttingen, Steinstr. 19, 37213 Witzenhausen, Germany; 3 National Institute of Animal Nutrition and Physiology (NIANP), Hosur Rd., Chennakeshava Nagar, Adugodi, Bengaluru 560030, Karnataka, India; 4 Rajiv Gandhi Institute of Veterinary Education and Research, Pondicherry 605008, India

**Keywords:** adaptive physiological responses, climatic challenges, dairy cattle, gene expressions

## Abstract

**Simple Summary:**

The effects of seasonal transition and temperature humidity index (THI) on a number of variables representing physiological, hematological and molecular responses of lactating dairy cows reared under natural environmental conditions were assessed. The study revealed significant impact of seasonal transition and THI on most of these variables. The physiological, hematological and molecular alterations highlight adaptive responses of bovine traits to climatic stressors. The results of this study contribute to a deeper understanding of the adaptive mechanisms of dairy cows under challenging environmental conditions, indicating their potential as heat stress biomarkers.

**Abstract:**

A comprehensive study was conducted to assess the effects of seasonal transition and temperature humidity index (THI) on the adaptive responses in crossbred dairy cows reared in a tropical savanna region. A total of 40 lactating dairy cattle reared by small-scale dairy farmers in Bengaluru, India, were selected for this study. The research period comprised the transitioning season of summer to monsoon, wherein all traits were recorded at two points, one representing late summer (June) and the other early monsoon (July). A set of extensive variables representing physiological responses (pulse rate, respiration rate, rectal temperature, skin surface temperature), hematological responses (hematological profile), production (test day milk yield, milk composition) and molecular patterns (PBMC mRNA relative expression of selective stress response genes) were assessed. A significant effect of seasonal transition was identified on respiration rate (RR), skin surface temperature, mean platelet volume (MPV), platelet distribution width (PDWc), test day milk yield and on milk composition variables (milk density, lactose, solids-not-fat (SNF) and salts). The THI had a significant effect on RR, skin surface temperature, platelet count (PLT), plateletcrit (PCT) and PDWc. Lastly, THI and/or seasonal transition significantly affected the relative PBMC mRNA expression of *heat shock protein 70* (*HSP70*), *interferon beta* (*IFNβ*), *IFNγ*, *tumor necrosis factor alpha* (*TNFα*), *growth hormone* (*GH*) and *insulin-like growth factor-1* (*IGF-1*) genes. The results from this study reveal environmental sensitivity of novel physiological traits and gene expressions to climatic stressors, highlighting their potential as THI-independent heat stress biomarkers.

## 1. Introduction

Environmental conditions and management practices modulate animals’ growth, production and welfare and these factors can be impaired due to climatic fluctuations. In parallel to the continually rising global milk demand, the dairy sector is adversely affected by climate change and its associated factors such as heat waves, droughts and solar flares [1]. In combination with the current economic crisis, this leads to inflated production costs and reduced milk production and export. Asia, Australia, Latin America, New Zealand and the European Union are among the regions that are mainly affected [2]. Nearly 60% of the total Asian population is involved in the agri-livestock sector with India being the largest milk producer and accounting for 19% of the global milk production [1]. Dairy farming in India and in most of the other Asian countries is dominated by small livestock holdings or mixed farms (87%), and only 10–12% are intensive operations [1,3]. This makes the Asian dairy sector vulnerable to climate change. Additionally, mathematical modeling analysis estimated the potential annual loss in milk production in the Trans and Upper Gangetic plains region of India due to heat stress for the time slice 2010–2039 which was equivalent to INR 11.93–12.44 billion based on two approaches [4]. 

Lactating cows are more predisposed to heat load when compared to dry cows and/or beef cattle as they have higher metabolic turnover [5,6]. Animals possess a number of phenotypic and genotypic characteristics that contribute to their adaptation to harsh environmental conditions. Adaptive mechanisms can be broadly categorized as morphological, behavioral, physiological, blood biochemistry, metabolic, neuro-endocrine and molecular and cellular responses [7]. The initial physiological mechanism adopted by dairy cattle in response to increasing temperature and humidity is heat dissipation from their bodies via evaporative cooling mechanisms [8]. Increased respiration rate and sweating enhance respiratory and cutaneous cooling mechanisms to dissipate heat during stressful thermal conditions [7]. However, the evaporative cooling mechanisms can become ineffective when temperature and humidity exceed a certain level, causing hyperthermia. At this point, other mechanisms to reduce heat load are activated, inducing alterations in metabolic activity and impaired productive performance [8,9]. 

Molecular responses to climatic stressors are activated at a late stage, when all the previously stated adaptive mechanisms are inadequate to revive homeostasis [10]. The responses to environmental stress, especially heat stress, involve a highly conserved cascade of altered gene expression and protein activation [11]. Most molecular studies on heat stress in livestock focus on the heat shock transcription factor (HSF) and heat shock protein (HSP) regulation as the gene expression component [12,13,14]. This is mainly due to the central cytoprotective role played by the HSPs during heat stress, thereby contributing to maintaining cellular integrity and homeostasis [10]. With increasing scientific interest in this field, researchers have been exploring the expression profile of several genes that might play a role during heat stress in cattle [15,16]. Some of these genes include *interleukin-1 beta* (*IL-1β*), *IL-6*, *tumor necrosis factor alpha* (*TNFα*), *B-cell lymphoma 2* (*BCL2*), *mechanistic target of rapamycin kinase* (*MTOR*), *signal transducer and activator of transcription 5B* (*STAT5B*), *insulin-like growth factor-1* (*IGF-1*) and *prolactin receptor* (*PRLR*) [15,16].

Climatic challenges will increase in the near future [17]. Though there are a number of indigenous cattle breeds that are well known for their thermo-tolerance, extensive crossbreeding practices contributed to the dilution of the respective germplasm [1]. Furthermore, there is a research gap in assessing the impact of climatic variation on dairy cattle and their adaptive responses. This is especially the case for cattle kept in environmentally challenging production systems, especially in countries such as India that are dominated by small-scale farming practices. Thus, the aim of the present study was to monitor the physiological, hematological and molecular responses of lactating crossbred cows in Bengaluru, India, in relation to seasonal transition and the temperature humidity index (THI).

## 2. Materials and Methods

### 2.1. Study Location and Animal Management

The study was conducted in one of the rising megacities in south India, Bengaluru (12.97° northern latitude and 77.59° eastern longitude, 920 m above sea level), which experiences tropical savanna climatic conditions. A total of 40 crossbred dairy cows (Holstein Friesian (HF) and Jersey breeds crossed with local breeds at varied proportions: HF cross = 19 cows; Jersey cross = 3 cows; HF–Jersey cross = 1 cow; mix of HF, Jersey and local breeds = 17 cows, reared by small-holder dairy farmers (12 farms) across Bengaluru were selected for the study. All selected cows were lactating and were reared in their respective farms according to the farmers’ usual feeding and management practices. Though their major dietary component consisted of green forage, all cows were also fed with 1.9–8.8 kg of concentrate feed per day.

### 2.2. Determination of the Temperature Humidity Index

The meteorological parameters, temperature and humidity, were recorded on the study farms using VOLTCRAFT DL-121TH USB data loggers (https://www.voelkner.de/products/270541/VOLTCRAFT-DL-121TH-Multi-Datenlogger-Messgroesse-Temperatur-Luftfeuchtigkeit-40-bis-70C-0-bis-100-rF.html, accessed on 1 December 2022) which were installed prior to trait recording. The data loggers recorded the temperature and humidity at an hourly interval. The indicator to assess heat stress in livestock, the THI, was calculated using the following equation from the National Research Council [18]:

THI = (1.8 × T + 32) − (0.55 − 0.0055 × RH) × (1.8 × T − 26)(1)

where T is air temperature in degrees Celsius and RH is relative humidity in percent.

For further analysis, the average value of all THI recordings during the seven days prior to the test day (day when the traits were recorded) (THI_1–7) was calculated and in agreement with a previous study [19], categorized in two classes, namely THI < 75 and THI ≥ 75.

### 2.3. Collection of Blood Samples and Hematological Analysis

Blood was taken from the jugular vein of the cows at two points in time that covered the environmental conditions late summer (June) and early monsoon (July) at an interval of 30 days. Approximately 7 mL of blood was collected from each cow in the morning before feeding, using sterile needles and EDTA coated vacutainers. The collected blood samples were placed in an icebox and transported to the laboratory to conduct further analyses. All hematological parameters were determined using the BC2800 autohematological analyzer (Mindray, Shenzhen, China). The following hematological parameters were determined: white blood cells (WBCs), lymphocytes, monocytes, granulocytes, red blood cells (RBCs), hematocrit (HCT), red cell distribution width (RDWc), platelet count (PLT), mean platelet volume (MPV), platelet distribution width (PDWc) and plateletcrit (PCT).

### 2.4. Physiological Trait Recording

All bovine traits were recorded twice, during the two seasons. Physiological trait recording in the field included pulse rate (PR), respiration rate (RR), rectal temperature (RT) and infrared thermography (IRT) of different body regions. The recorded production trait from the same test date was test day milk yield and the lactation stages of the cows were also noted. Additionally, approximately 30 mL of milk was collected from each cow to assess the milk composition in the laboratory. All traits were recorded by the same experienced person. 

**Figure 1:**
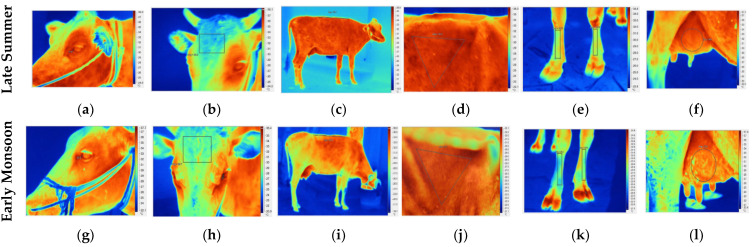
Representative IRT images captured during the late summer (**a**–**f**) and early monsoon (**g**–**l**) seasons of different body parts; eye (**a**,**g**), forehead (**b**,**h**), back (**c**,**i**), flank (**d**,**j**), foreshank (**e**,**k**) and udder (right fore quarter, (**f**,**l**)).

The PR was recorded by palpating the coccygeal artery located at the ventral surface of the tail head for one minute, reflecting the number of beats per minute. The RR was recorded by observing the flank movement at a distance of several meters to avoid disturbing the cow’s natural behavior. The RR was expressed as breaths per minute. A digital rectal thermometer was used to record RT in degrees Celsius (°C). The surface temperature was recorded using non-invasive infrared thermography (IRT) of the following body regions (Figure 1): eye, forehead, back, flank, foreshank and udder (right fore quarter (RFQ), left fore quarter (LFQ), right hind quarter (RHQ), left hind quarter (LHQ)). To carry this out, we used a handheld digital thermal imaging infrared camera (TI200–9 Hz, image resolution: 200 × 150 pixels; Fluke Corporation, Everett, WA, USA; emissivity set 0.98). Each IRT image was taken at a distance of approximately 1.0 m from the body location. All IRT images were interpreted using the Fluke Smartviewer™ 4.0 (Fluke Corporation, Everett, WA, USA) software and the final parameters were expressed in degrees Celsius (°C). 

Milk composition traits included density, lactose, solids-not-fat (SNF), protein and salts, which were determined using the Lactoscan Milk Analyzer (Softrosys Technologies, Bengaluru, India) following the manufacturer’s instruction.

### 2.5. Total mRNA Extraction and Complementary DNA (cDNA) Synthesis

Work pertaining to mRNA extraction and quantitative real-time PCR (qPCR) analyses was carried out using the MIQE guidelines that describe the minimum information necessary for evaluating qPCR experiments [20]. The work surface of the laboratory was wiped with 70 percent ethanol. On the day of isolation, the UV lamp of a laminar flow biosafety cabinet was switched on for 20 min prior to the processing of samples. The plastic wares used were certified by manufacturers to be RNase free. RNaseZAP (Sigma-Aldrich) was used to clean the gloves and work surface to ensure a RNase-free environment for RNA isolation.

The mRNA extractions from the blood samples were processed on the day of sample collection, immediately after reaching the laboratory. The peripheral blood mononuclear cells (PBMCs) were isolated using the RBC lysis method wherein ice-cold 1× RBC lysis buffer was added to 5 mL of freshly collected blood samples at a ratio of 9:1, and incubated at room temperature for 15–20 min. Afterwards, the samples were centrifuged at 3500 rpm for 20 min. The supernatant containing lysed erythrocytes was discarded. The pellet was re-suspended in RBC lysis buffer and the above steps were repeated until a clear pellet was obtained. The obtained PBMC pellet was then processed for mRNA extraction using the GeneJET Whole Blood RNA Purification Mini Kit (Thermo Scientific, Vilnius, Lithuania) following the manufacturer’s protocol with minor modifications. On completing the extraction, the RNA pellet was eluted in nuclease-free water and stored at −80 °C. The RNA concentration was measured by using a spectrophotometer (NanoDrop^TM^ 2000 C) and all samples with positive quality check were converted to complementary DNA (cDNA). The cDNA was synthesized using a RevertAid First Strand cDNA Synthesis Kit (Thermo scientific, Vilnius, Lithuania) and was stored at −80 °C until further use.

### 2.6. Quantitative PCR 

Selective genes that were reported to be associated with stress in ruminants were selected for the analysis of gene expressions. The genes were broadly grouped as genes associated with (i) adaptation: *heat shock factor-1* (*HSF1*), *heat shock protein 70* (*HSP70*) and *HSP90*; (ii) inflammatory/immune response: *interleukin 18* (*IL18*), *interferon gamma* (*IFNγ*), *IFNβ* and *tumor necrosis factor alpha* (*TNFα*); (iii) production: *growth hormone* (*GH*), *growth hormone receptor* (*GHR*), *insulin-like growth factor-1* (*IGF-1*) and *leptin* (*LEP*). Bovine-specific primers to amplify the short fragment of each target gene and endogenous reference genes *glyceraldehyde-3-phosphate dehydrogenase* (*GAPDH*) and *hypoxanthine phosphoribosyl transferase 1* (*HPRT*) were designed from previously published literature. The detailed primer sequences with their respective references are depicted in Supplementary Table S1. Quantitative PCR was performed using SYBR Green Chemistry (Maxima SYBR Green qPCR Master Mix, Fermentas, Waltham, MA, USA). The data obtained for each of the target genes (cycle threshold, Ct) were normalized to the geometric mean of the two reference genes, *GAPDH* and *HPRT*, to obtain the ΔCt value (target gene Ct − mean reference gene Ct = ΔCt). This value was then used for the statistical analysis, and the *2^−ΔΔCT^* method [21] was used to obtain the relative expression results for visual representation. The relative expression of each gene, during late summer and in THI class 2 (THI ≥ 75), was compared, respectively, to that of early monsoon and THI class 1 (THI < 75), and the results were expressed as fold change. 

### 2.7. Statistical Analysis

All the data collected were analyzed by applying SAS University Edition (SAS/STAT^®^, SAS Institute Inc., Cary, NC, USA). In this regard, for the repeated measurements per trait, we applied the MIXED procedure to infer fixed and random effects via mixed model equations. The statistical model was:

Y_ijklmn_ = μ + LS_i_ + F_j_ + THI_k_ + S_l_ + A_m_ + e_ijklmn_ (2)

where Y_ijklmn_ was the observation for the production, hematological, physiological and gene expression (ΔCt value) traits from the ijklmn th cow, μ was the overall mean effect, LS_i_ was the fixed effect for the ith lactation stage class (i ≤3 months, 3–9 months, >9 months), F_j_ was the fixed effect for the jth farm, THI_k_ was the fixed effect for the kth THI_1–7 class (average of THI recordings throughout the seven days prior to test day; with the two classes THI < 75 or THI ≥ 75) for production, hematological and physiological traits, and the test day THI for the gene expression traits (with two classes THI < 75 or THI ≥ 75) as suggested for these kinds of data [22], S_l_ was the fixed effect for the lth season (l= late summer, early monsoon), A_m_ was the random cow effect for two repeated measurements of the mth animal and e_ijklmn_ was the random residual effect.

## 3. Results

### 3.1. Temperature Humidity Index

The descriptive statistics for the overview of the monthly average ambient temperature, humidity and THI during the two collection months (June: late summer; July: early monsoon) are depicted in Supplementary Table S2. An average monthly ambient temperature of 25.99 ± 2.66 °C (Min: 20.1 °C; Max: 36.5 °C), relative humidity of 75.18 ± 8.94% (min: 42.9%; max: 93.9%) and THI of 75.77 ± 3.32 (min: 67.43; max: 86.92) were recorded during June. The monthly average weather variables recorded during July were as follows: ambient temperature 25.06 ± 2.37 °C (min: 19.7 °c; max: 33.8 °C); relative humidity 78.37 ± 7.84% (min: 43.6%; max: 93.1%); THI 74.71 ± 3.17 (min: 66.66; max: 84.35).

### 3.2. Physiological Response and Infrared Thermography

The descriptive statistics for all the traits recorded throughout the study period are depicted in Supplementary Table S3. Table 1 and Table 2 depict the least square means for the physiological response variables and surface temperature recorded with infrared thermography.

The THI effect was non-significant on PR, RR and RT. The seasonal transition significantly (*p* < 0.05) affected RR, with a significantly higher RR during late summer (29.66 ± 1.17 breaths/min) when compared to early monsoon (23.44 ± 1.93 breaths/min). 

For all body surface temperatures recorded via IRT, a significant (*p* < 0.01) influence of THI (except on eye and forehead) and seasonal transition was observed (Table 2). Surface temperatures from all body regions were significantly (*p* < 0.01) higher in the higher THI class (THI ≥ 75). With 32.21 ± 0.27 °C, 34.12 ± 0.19 °C and 28.74 ± 0.25 °C, respectively, the least square means for surface temperature recorded on the back, flank and foreshank regions under THI ≥ 75 were significantly higher (*p* < 0.01) than corresponding values at THI < 75 (29.92 ± 0.42 °C for the back, 32.56 ± 0.30 °C for the flank, 26.39 ± 0.38 °C for the foreshank region). A similar trend was noticed for the seasonal influence on surface temperatures for all body regions. The least square means for IRT readings during the late summer for eye (37.34 ± 0.05 °C), forehead (30.69 ± 0.18 °C), back (32.27 ± 0.20 °C), flank (34.53 ± 0.14 °C) and foreshank (29.00 ± 0.18 °C) were significantly higher (*p* < 0.01) than the respective values recorded during early monsoon (36.88 ± 0.09 °C, 29.41 ± 0.31 °C, 29.86 ± 0.34 °C, 32.15 ± 0.24 °C and 26.12 ± 0.31 °C). Similarly, the udder surface temperature was also significantly higher (*p* < 0.01) for all quarters during the late summer season and THI ≥ 75 when compared to early monsoon and THI < 75.

### 3.3. Hematological Profile

None of the leukocyte (WBC, lymphocyte, monocyte and granulocyte) and erythrocyte (RBC, HCT and RDWc) indices were significantly influenced by THI and/or season (Supplementary Table S4). The thrombocyte indices (PLT, MPV, PDWc and PCT) were significantly affected by THI and/or seasonal transition (Table 3). 

A highly significant (*p* < 0.01) effect of THI was observed on PLT, PDWc and PCT. The PLT concentration was significantly higher (*p* < 0.01) with 440.10 ± 51.89 × 10^3^ µL at a THI < 75 compared to 132.12 ± 37.23 × 10^3^ µL at a THI ≥ 75. Likewise, a significant decline in PCT was observed at THI ≥ 75 (0.08 ± 0.02%) when compared to THI < 75 (0.24 ± 0.03%). The PDWc depicted an opposite trend, wherein higher values were observed in the higher THI class (THI ≥ 75: 17.51 ± 0.17% vs. THI < 75: 16.51 ± 0.24%). Lastly, the seasonal transition had a significant (*p* < 0.05) effect on MPV and PDWc, both depicting lower values during early monsoon (MPV: 5.25 ± 0.19 fL; PDWc: 16.77 ± 0.19%) when compared to late summer (MPV: 5.80 ± 0.11 fL; PDWc: 17.26 ± 0.12%). 

### 3.4. Milk Performance and Composition

Table 4 depicts the least square means for test day milk yield and milk composition.

The least square means for test day milk yield significantly differed across the two collection seasons (*p* < 0.05). A significantly lower test day milk yield was observed during the late summer (9.15 ± 0.76 L/day) than in early monsoon (13.15 ± 1.34 L/day). Lactation stage also significantly (*p* < 0.01) influenced the test day milk yield in crossbred dairy cows with higher milk yield at early lactation stages (lactation stage < 3 months: 15.49 ± 1.34 L/day, 3–9 months: 10.19 ± 0.85 L/day and > 9 months: 7.77 ± 1.53 L/day). The THI_1–7 was not significant for test day milk yield. 

The milk composition revealed a significant (*p* < 0.05) seasonal effect on milk density, lactose, SNF and salts. All these variables were significantly lower (*p* < 0.05) during early monsoon than in late summer. Furthermore, the farm effect significantly (*p* < 0.05) affected all milk composition variables, while the THI_1–7 effect was not significant for any milk composition variables. 

### 3.5. Selective Gene Expression Analysis

**Figure 2:**
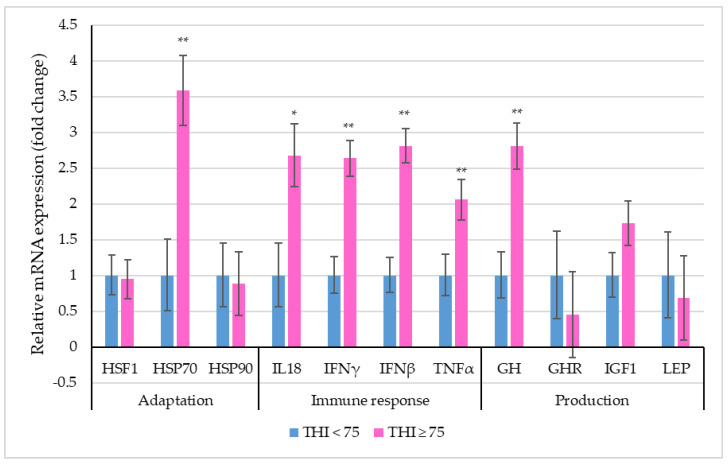
Relative mRNA expression (the vertical lines indicate standard errors) of selective genes in PBMCs of dairy cows within two THI classes (* *p* < 0.05; ** *p* < 0.01).

Figure 2 depicts the relative mRNA expression of selective stress-associated genes in PBMCs of dairy cattle between the two THI classes.

The THI significantly influenced the relative expression profile of *HSP70*, *IL18*, *IFNγ*, *IFNβ*, *TNFα* and *GH* genes in crossbred cows. A 3.58-fold increase in the PBMC mRNA expression of *HSP70* was observed for THI ≥ 75 as compared to THI < 75. All the immune response genes were observed to be up-regulated in the higher THI class. The relative expression profiles of *IL18* (*p* < 0.05), *IFNγ*, *IFNβ* and *TNFα* differed significantly (*p* < 0.01), exhibiting a 2.68-fold, 2.64-fold, 2.81-fold and 2.06-fold increase under THI ≥ 75 in relation to THI < 75. Lastly, among the production-related genes, the relative expression profile of *GH* was 2.81-fold higher (*p* < 0.01) at THI ≥ 75 when compared to THI < 75.

**Figure 3:**
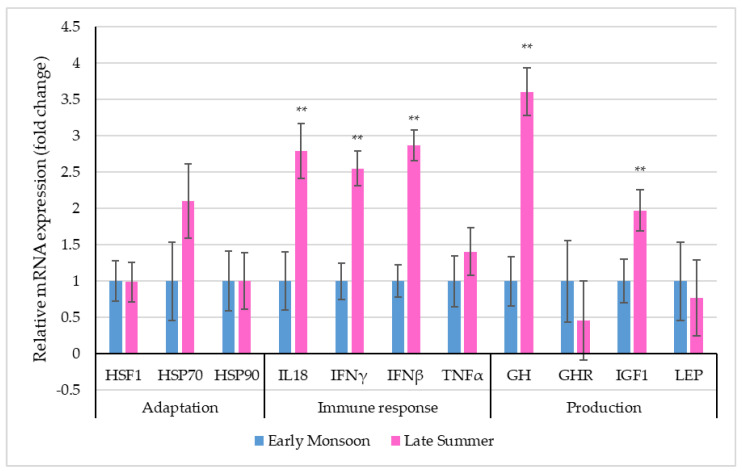
Relative mRNA expression (the vertical lines indicate standard errors) of selective genes in PBMCs of dairy cows in two transitioning seasons (** *p* < 0.01).

Figure 3 depicts the relative mRNA expression of selective stress-associated genes in PBMCs of dairy cattle during the two transitioning seasons.

Though the relative expression profiles of the adaptation-related genes, i.e., *HSF1, HSP70* and *HSP90*, revealed altered expression levels, differences between seasons were non-significant (*p* > 0.05). Among the immune response-related genes, the relative expression profiles of *IL18*, *IFNγ* and *IFNβ* were significantly (*p* < 0.01) altered due to seasonal transition. Significantly up-regulated PBMC mRNA expressions of *IL18* (2.79 ± 0.38-fold), *IFNγ* (2.55 ± 0.24-fold) and *IFNβ* (2.87 ± 0.21-fold) were observed during late summer when compared to early monsoon. Among the production-related genes, the relative expression of *GH* (*p* < 0.01) and *IGF1* (*p* < 0.01) was significantly up-regulated during late summer (*GH*: 3.61 ± 0.33-fold; *IGF1*: 1.97 ± 0.01-fold) when compared to early monsoon. 

## 4. Discussion

In tropical countries experiencing hot and humid seasons, the seasonal changes adversely affect livestock production, reproduction, immunity and welfare. A rise in environmental temperature and humidity implies stress for the animals, altering their cardinal physiological responses both directly and indirectly. The impacts are perceptible by altered physiological reactions, behavior, reduced feed intake, lowered growth rate, decreased milk production and increased occurrence of disease outbreaks [24,25,26]. There is a lack of studies monitoring the adaptive responses of dairy cattle reared in small-scale farming systems. The results from the present study contribute to filling this gap by providing insights into the adaptive responses of crossbred dairy cattle in Bengaluru during the seasonal transition from hot, dry summer to monsoon. The present study also infers the effects of THI on physiological, hematological and molecular responses.

### 4.1. Physiological Response and Infrared Thermography

In a study by Rhoads et al. [27], reduced feed intake accounted for only 35% of the heat stress-induced reduction of milk yield in cows, while the remaining part was due to alterations in the somatotropic axis inducing a cascade of physiological processes. Physiological response is a vital adaptive mechanism of animals subjected to climatic stress. The RR, RT, PR, skin surface temperature and sweating rate are some of the classical indicators to assess thermal adaptability/susceptibility in animals. In the present study, a significant impact of THI on RR and all the IRT traits (except for eye and forehead IRT) was identified, while season significantly influenced only the IRT measurements. Though most of the physiological variables observed during the study were almost within the normal range, their differences between seasons and THI classes are noteworthy. Respiration rate is suggested as an ideal biomarker for climatic stress in livestock. Enhanced RR during thermal stress indicates activation of respiratory evaporative cooling in animals [9]. The increasing RR with rising THI as observed in the current study might reflect the activation of this adaptive mechanism in dairy cows. Kumar et al. [26] evaluated the dynamics of varied heat stress responses in lactating Hariana cows at different THI levels. RR and PR were significantly increased at THI 78 and 80. Along with a significant alteration at the physiological level, the authors also reported altered production (decreased milk yield), as well as metabolic and molecular responses in Hariana cows with alterations in THI. Furthermore, a significant influence of lactation stage was observed on RR, with highest values for RR for cows in the early lactation stage (0 to 3 months). This could be due to the additional effect of production energy demand, since peak milk production in dairy cows is usually observed during the early lactation stage [28]. In a recent report by Yan et al. [29], significantly higher RT and RR were observed in early lactation cows when compared to those in late lactation, for thermo-neutral as well as for heat stress conditions.

Sweating rate, sweat gland characteristics and skin surface temperature are among the indicators that reflect cutaneous evaporative cooling, another important adaptive response of animals under thermal stress [9,30]. Infrared thermography and production of thermal color maps is an accurate and non-invasive method with wide applicability and is gaining increasing attention in assessing thermal stress [31,32]. In the current study, the surface temperatures of different body regions were significantly influenced by THI and season. Differences of almost 1 to 3 degrees Celsius in IRT measurements were observed in cows between seasons and between THI classes, with higher skin temperature recorded in the higher THI class (THI ≥ 75) and in the warmer season (late summer). A similar increase in body surface temperature with increasing THI was reported by Peng et al. [33] in lactating Chinese Holstein cows. These authors also observed that the body surface temperature seems to be more sensitive to thermal stress than RT. Furthermore, studies also reported variations in skin temperature across different body regions in animals, reflecting varied heat dissipation abilities [33,34,35]. In the present study, the eye IRT recorded the highest surface temperature, followed by udder, flank, back, forehead and lastly foreshank. Kim et al. [36] also reported the highest IRT readings in the eye and hindquarter regions, when compared to measurements from the nose, ear and parts of the horn. In addition, the authors also reported that the IRT measurements from eyes and hindquarters displayed greater stability throughout the year. 

It is interesting to note that RT, which again is a well-established physiological marker for heat stress, was not significantly altered due to season or THI alterations in the present study. This could be due to the fact that the climatic stress experienced by the animals was of a lower magnitude than that needed to induce changes in their RT. When an animal is subjected to an environment above the upper critical limits (can range from 24–27 °C), the first mechanism to be activated is evaporative cooling (respiratory cooling and sweating) [37,38,39]. On exposure to extremely harsh conditions, wherein the previously stated evaporative mechanisms were not sufficient to maintain homeothermy, the RT increased above the normal upper limit (39.3 °C) [38,40]. In the present study, RR and IRT were significantly altered due to THI and/or seasonal transition, but RT remained unaffected. Since all traits were recorded during the morning hours with cooler climatic conditions compared to peak afternoon periods, the studied crossbred cows were seemingly able to cope with the relatively lower thermal stress by activating regular cooling mechanisms (increasing RR and altered IRT) to effectively regulate RT. 

### 4.2. Hematological Profile

The hematological profile is another crucial indicator depicting the physiological status of an animal. Though the hematological profile is influenced by several factors, the variations caused due to climatic factors (THI and seasonal variations) indicate physiological responses to stress, welfare and adaptation [41,42,43]. In the present study, most of the hematological variables were within the previously established reference range limits. However, some values of the thrombocyte indices (PLT and PCT) exceeded the reference range. The significantly higher PLT value at THI < 75 is still within the acceptable limits [44,45]. PLT and PCT values below the reference range definitely indicate low platelet counts in crossbred cattle. Climatic variations (THI and/or seasonal variation) significantly altered the thrombocyte indices (PLT, MPV, PDWc and PCT) in dairy cows. The PLT and PCT were significantly lower in the higher THI class and during the summer season, while MPV and PDWc displayed an opposite pattern. Thrombocytes are known for their role in aggregation and clot formation, and they are vital mediators of innate and adaptive immunity [46,47]. In a study by Casella et al. [42], lactating Bruna cows showed the lowest PLT and PCT values during months with the highest THI. Other studies also indicated decreased platelet counts in cattle with increasing environmental temperature and/or during hot seasons [42,43,48]. Thrombocytopenia during harsh thermal conditions was associated with hemodilution, thrombopoietic suppression and increased platelet aggregation, as well as increased consumption and destruction of platelets [47,49]. However, the thrombocytic indices observed in the present study—high MPV and PDW—along with low PLT and PCT during high THI and/or summer are pointing to megakaryocyte responses to low platelet count by rapid production and release of large platelets into the circulation [47,50]. Such mechanisms can be interpreted as an adaptive response to heat stress exhibited by the studied crossbred dairy cows. 

None of the leucocyte and/or erythrocyte indices were significantly influenced by THI and seasonal transition. Though some of these variables were associated with climatic stress, their non-significance could be due to the short study period, variations in cow water intake (which was not possible to record under practical field conditions) or due to the animals’ innate ability to activate alternative adaptive mechanisms. Similar non-significant influence of short-term heat stress on blood hematological profile was reported by Jo et al. [51] for lactating Holstein cows of Korea. 

### 4.3. Milk Performance and Composition

In the present study, test day milk yield was numerically lower at the higher THI level. In addition, test day milk yield recorded during late summer was significantly lower than that during the early monsoon. This could be attributed to several factors, with reduced feed intake upon exposure to climate stress and its associated negative energy balance being one probable cause [52]. In a previous study conducted in the same location, season and THI significantly affected test day milk yield of exotic and crossbred dairy cows [19]. Moreover, the highest milk yield was reported during the rainy season, followed by summer and winter seasons [19]. Milk composition is a crucial variable as it influences the down-stream processing of milk. A significant influence of seasonal transition on milk composition was also observed in the current study. In early monsoon, a significant decline in milk density, lactose, SNF and salts was observed in crossbred cows as compared to late summer, which is partly explained by a dilution effect of the higher milk yield. Seasonal variations in milk density and milk composition of Holstein Friesian cows from Ireland were presented by Parmar et al. [53], where higher density values (1.0314 g/cm^3^) were observed during the summer season (June–August). Sharma et al. [54] reported a significant seasonal influence on milk composition of crossbred cattle reared in the Sub-Himalayan Region of north India, with a significant decline in SNF and total solids during the summer season (April–June) when compared to the rainy season (July–September). In a study by Veena et al. [55] with crossbred cattle from Punjab, north India, milk protein and SNF were lower during the winter season (November–February) than during summer (May–August). Another possible factor contributing towards altered milk yield and composition is water intake [54] as well as a change in diet composition.

### 4.4. Selective Gene Expression Analysis

*HSF* and *HSPs* are important genes associated with thermal stress, also known as molecular chaperones. They play a crucial cytoprotective role during cellular stress by preventing the formation of non-functional proteins and maintaining protein folding [26,56]. Numerous studies assessed the expression profile of these genes, especially *HSP70*, *HSP90* and *HSF1*, in response to varying stressors including seasonal variation and THI [56,57]. In a study by Collier et al. [22], it was observed that the expression of *HSP70* in bovine mammary epithelial cells remained elevated for 4 h after exposure to heat stress and returned to basal levels after 8 h. This was also one of the reasons for using the test day THI in the model while assessing the statistical significance for the gene expression analysis. Significant up-regulation of key HSPs (i.e., *HSP40*, *HSP60*, *HSP70* and *HSP90*) along with *HSF1* was reported in indigenous Sahiwal and Kankrej cows of the Thar Desert during the hotter seasons (summer and hot–humid) [56]. Likewise, Gill et al. [58] also stated a nearly one- to threefold increase in PBMC mRNA expression of the *HSF1* gene in heat stressed Sahiwal and crossbred cows. In the present study, among the three adaptation-related genes (*HSF1, HSP70* and *HSP90*), the relative expression of *HSP70* was up-regulated in the higher THI class (THI ≥ 75) and late summer season (non-significant). *HSP70* is a vital isoform among the heat shock group of proteins that can provide an immediate relief from heat stress challenges [13]. The early induction of this molecular chaperone during thermal stress was also reported to provide a “second window of protection” [59] that can protect against cellular stress. Such an early induction of *HSP70* could also be associated with the development of thermal tolerance in animals [56,60]. Apart from their potential cytoprotective activity, HSPs also activate the immune response mechanisms during heat stress [11].

Another potential consequence of climatic stress is impaired immunity. Though there are an appreciable number of studies assessing the impact of seasonal variation and heat stress on physiological responses of dairy cattle, the association with immune response still needs to be extensively evaluated. Inflammatory cytokines such as TNFα, interleukins and interferons play a significant role in stimulating systemic inflammatory responses, which were mostly up-regulated during stress [16,61,62]. In the current study, all of the selected immune response-associated genes, *IL18*, *IFNγ*, *IFNβ* and *TNFα*, were up-regulated at THI ≥ 75 and during the late summer season (although *TNFα* was non-significant) when compared with THI < 75 and early monsoon, respectively. Certain studies have reported similar alteration in the expression profile of immune response-related genes in cattle. Grewal et al. [16] observed significantly higher PBMC mRNA expressions of *IL-1β*, *IL-6*, *IL-10* and *TNF-α* in Sahiwal cows during the summer season when compared to winter. In another study, significantly higher expression of PBMC inflammatory cytokines was observed in heat stressed cows in comparison to cows kept under cooling conditions during the transition period [63]. Likewise, up-regulation of *TNF-α*, *IL-1β*, *IL6* and *IL-10* was reported by Kapila et al. [64] when exposing bovine mammary epithelial cells to heat stress. Furthermore, Chen et al. [65] reported an increase in pro-inflammatory cytokines in heat stressed dairy cows. These authors also stated a possible role of the brain–gut axis in heat stressed dairy cows that modulates their physiology and immunity, possibly by interacting with the animals’ rumen microbial activity leading to altered cytokine levels. 

Growth hormone (GH) and insulin-like growth factor-1 (IGF-1) are members of the somatotropic axis that play a crucial role in regulating metabolism and growth physiology in mammals. The production of these hormones is influenced during heat stress in cows because of the negative energy balance in response to reduced feed intake [66,67]. Though there are a number of reports assessing the influence of *GH* and *IGF1* genes on growth [68], lactation [69] and reproduction [70] in cattle, there are sparse reports assessing the seasonal and THI impact on their expression. In the present study, the relative PBMC mRNA expressions of *GH* (3.60-fold) and *IGF1* (2.03-fold) were significantly up-regulated during late summer when compared to early monsoon. The circulating GH levels can increase when the animal is in a negative energy balance [11]. Therefore, the significant up-regulation of *GH* as observed in the present study could highlight a possible stressful situation in dairy cows during harsh climatic conditions as well as the lactation-induced nutrient requirements. A study on heat stressed goats also reported increased plasma GH levels [71]. Tao et al. [63] reported increased PBMC *IGF1* mRNA expression in heat stressed cows which plays a role in enhancing the expression of inflammatory cytokine genes through a paracrine or autocrine pathway. A similar result of increased relative expression of cytokine genes was identified in the present study, too.

## 5. Conclusions

The present study revealed a significant impact of seasonal transition and THI on major physiological, hematological and molecular responses of crossbred dairy cows. Though the influence of season and/or THI on most of the studied variables has been established previously, studies considering all these variables under extremely harsh on-farm conditions of smallholder farmers are barely reported. As evident from the results, RR and skin surface temperature were identified as the most valuable indicators for climatic stress in dairy cattle. Additionally, the hematological and relative gene expression profiles point towards activated inflammatory/immune responses in dairy cows due to climatic stress. The THI and/or season significantly influenced the relative expression of *HSP70*, *IL18*, *IFNγ*, *IFNβ*, *TNFα*, *GH* and *IGF1* genes. Furthermore, the reduced milk yield and altered milk composition during the summer season calls for appropriate mitigation measures, such as cooling possibilities and adjusted feeding, to reduce productive losses for the livestock breeders. Extending such studies to diverse dairy cattle breeds and coupling the analysis of physiological and molecular heat stress responses with genome-wide association studies may be a promising tool to identify breeds with a high tolerance of heat stress—a characteristic of increasing relevance with accelerating global warming.

## Figures and Tables

**Table 1:** Test of significance (F-test) for the effects of the statistical model and least square means with standard errors for the effect levels of THI and season on physiological cow traits.

Effects	PR (Beats/min)	RR (Breaths/min)	RT (°C)
FarmID	NS	*	NS
THI_1–7	NS	NS	NS
1 (THI < 75)	69.19 ± 3.77	23.21 ± 2.38	37.14 ± 0.21
2 (THI ≥ 75)	70.02 ± 2.39	29.90 ± 1.59	37.37 ± 0.14
Season	NS	*	NS
Late summer	72.94 ± 1.87	29.66 ± 1.17	37.40 ± 0.11
Early monsoon	66.27 ± 3.08	23.44 ± 1.93	37.11 ± 0.17
Lactation stage	NS	NS	NS

NS: non-significant; * *p* < 0.05; PR: Pulse rate; RR: Respiration rate; RT: Rectal temperature.

**Table 2:** Test of significance (F-test) for the effects of the statistical model and least square means with standard errors for the effect levels of THI and season on infrared thermography of dairy cows.

Effects	Eye (°C)	Forehead (°C)	Back (°C)	Flank (°C)	Foreshank (°C)	Right Fore Quarter (°C)	Left Fore Quarter (°C)	Right Hind Quarter (°C)	Left Hind Quarter (°C)
FarmID	NS	NS	**	**	**	**	**	**	**
THI_1–7	NS	NS	**	**	**	**	**	**	**
1 (THI < 75)	36.99 ± 0.12	29.63 ± 0.39	29.92 ± 0.42	32.56 ± 0.30	26.39 ± 0.38	33.12 ± 0.26	33.24 ± 0.29	32.95 ± 0.28	32.87 ± 0.31
2 (THI ≥ 75)	37.24 ± 0.08	30.46 ± 0.25	32.21 ± 0.27	34.12 ± 0.19	28.74 ± 0.25	34.77 ± 0.17	34.68 ± 0.19	34.81 ± 0.18	34.90 ± 0.20
Season	**	**	**	**	**	**	**	**	**
Late summer	37.34 ± 0.05	30.69 ± 0.18	32.27 ± 0.20	34.53 ± 0.14	29.00 ± 0.18	34.85 ± 0.12	34.85 ± 0.14	34.76 ± 0.13	34.80 ± 0.14
Early monsoon	36.88 ± 0.09	29.41 ± 0.31	29.86 ± 0.34	32.15 ± 0.24	26.12 ± 0.31	33.04 ± 0.21	33.07 ± 0.24	33.00 ± 0.23	32.97 ± 0.25
Lactation stage	NS	NS	NS	NS	NS	NS	NS	NS	NS

NS: non-significant; ** *p* < 0.01.

**Table 3:** Test of significance (F-test) for the effects of the statistical model and least square means with standard errors of the effect levels for THI and season on thrombocyte indices of dairy cows.

Effects	PLT (×10³/µL)	MPV (fL)	PDWc (%)	PCT (%)
Overall mean	272.99 ± 137.30	5.70 ± 0.58	17.14 ± 0.69	0.15 ± 0.07
THI_1–7	**	NS	**	**
1 (THI < 75)	440.10 ± 51.89	5.29 ± 0.24	16.51 ± 0.24	0.24 ± 0.03
2 (THI ≥ 75)	132.12 ± 37.23	5.77 ± 0.16	17.51 ± 0.17	0.08 ± 0.02
Season	NS	*	*	NS
Late summer	240.17 ± 28.09	5.80 ± 0.11	17.26 ± 0.12	0.14 ± 0.01
Early monsoon	332.05 ± 45.46	5.25 ± 0.19	16.77 ± 0.19	0.17 ± 0.02
Lactation stage	NS	NS	NS	NS
Reference range [23]	151–359	5.3–7.2	15.6–18.4	0.12–0.25

NS: non-significant; * *p* < 0.05; ** *p* < 0.01; PLT: Platelet count; MPV: Mean platelet volume; PDWc: Platelet distribution width; PCT: Plateletcrit.

**Table 4:** Test of significance (F-test) for the effects of the statistical model and least square means with standard errors of the effect levels for THI and season on milk yield and milk composition in dairy cows.

Effects	Milk Yield (L/Day)	Density (kg/m^3^)	Lactose (%)	SNF (%)	Protein (%)	Salts (%)
FarmID	**	*	**	**	**	*
THI_1–7	NS	NS	NS	NS	NS	NS
1 (THI < 75)	11.93 ± 1.67	1022.85 ± 1.04	4.12 ± 0.11	7.50 ± 0.21	2.72 ± 0.09	0.62 ± 0.03
2 (THI ≥ 75)	10.38 ± 1.07	1022.80 ± 0.77	3.94 ± 0.07	7.16 ± 0.13	2.62 ± 0.05	0.62 ± 0.02
Season	*	*	*	*	NS	*
Late summer	9.15 ± 0.76	1023.82 ± 0.66	4.14 ± 0.06	7.52 ± 0.11	2.74 ± 0.04	0.64 ± 0.02
Early monsoon	13.15 ± 1.34	1021.83 ± 0.77	3.91 ± 0.07	7.15 ± 0.14	2.61 ± 0.06	0.60 ± 0.02
Lactation stage	**	NS	NS	*	*	NS

NS: non-significant; * *p* < 0.05; ** *p* < 0.01.

## Data Availability

The anonymized data set that forms the basis of this article is available through the institutional repository at the University of Göttingen. For scientific purposes, access will be provided upon written request to the corresponding author.

## Supplementary Materials

The following supporting information can be downloaded at: https://www.mdpi.com/article/10.3390/biology12010026/s1, Table S1: Details of primer sequences used for relative gene expression analysis; Table S2: Descriptive statistics depicting the overview of the mean weather variables during the months of June and July; Table S3: Descriptive statistics of all the physiological, hematological, production and gene expression traits recorded throughout the study period; Table S4: Least square means with standard errors for influence of THI and season on leukocyte and erythrocyte indices of dairy cows.
