# Supplementary material for: Molecular, Physiological and Hematological Responses of Crossbred Dairy Cattle in a Tropical Savanna Climate
Source: Biology (Basel). 2022 Dec 23;12(1):26. doi: 10.3390/biology12010026 (PMC9855086; doi:10.3390/biology12010026)
Supplement: Supplementary file 1 [file biology-12-00026-s001.zip › biology-2103981-supplementary.pdf]

Table S1. Details of primer sequences used for relative gene expression analysis.

Sr No	Gene		Sequence (5'- 3')	Product size	Reference
1.	HSF1	F	CCCCGACCACCCTTATTG	139	[73]
		R	GCGACGCTGAGGCACTT		
2.	HSP70	F	TACGTGGCCTTCACCGATAC	171	[74]
		R	GTCGTTGATGACGCGGAAAG		
3.	HSP90	F	GGAGGATCACTTGGCTGTCA	177	[74]
		R	GGGATTAGCTCCTCGCAGTT		
4.	IL18	F	CTATTGAGCACAGGCATAAAGATG	131	[75]
		R	TGATCTGATTCCAGGTCTTCATCA		
5.	IFNγ	F	TCAAATTCCGGTGGATGATCTGC	106	[75]
		R	TCTCTTCCGCTTTCTGAGGT		
6.	IFNβ1	F	CCAGGAGCTACAGCTTGCTT	121	NM_174350.1
		R	ATCTGGAAGTCCATCCTGGC		
7.	TNFα	F	TCTTCTCAAGCCTCAAGTAACAAGT	103	[76]
		R	CCATGAGGGCATTGGCATAC		
8.	GH1	F	GTTTGCCAACGCTGTGCT	118	[77]
		R	CTGGGTGTTCTGGATGGAGTA		
9.	GHR	F	CGTGGACAACGCTTACT	130	[78]
		R	AAGGGTTTCTGTGGTGAT		
10.	IGF1	F	GTTGGTGGATGCTCTCGAGT	148	[79]
		R	CTCCAGCCTCCTCAGATCAC		
11.	PRLR	F	GAACCTCAGGCCCATCCCT	65	[80]
		R	TCCGGATTCTCCAGCTTCTC		
12.	β-actin	F	TTAGCTGCGTTACACCCTT	159	[78]
		R	GTCACCTTCACCGTTCCA		
13.	HPRT	F	AGTCCGAGTTGAGTTTGGAA	187	[81]
		R	GGCTCGTAGTGCAAATGAAGAGT		
14.	GAPDH	F	GGCAAGGTCATCCCTGAG	90	[74]
		R	GCAGGTCAGATCCACAACAG		

Table S2. Descriptive statistics depicting the overview of the mean weather variables during the months of June and July.

Month	Ambient temperature (°C)			Humidity (%)			Temperature Humidity Index		
	Mean ± SD	Min	Max	Mean ± SD	Min	Max	Mean ± SD	Min	Max
June	25.99 ± 2.66	20.1	36.5	75.18 ± 8.94	42.9	93.9	75.77 ± 3.32	67.43	86.92
July	25.06 ± 2.37	19.7	33.8	78.37 ± 7.84	43.6	93.1	74.71 ± 3.17	66.66	84.35

Table S3. Descriptive statistics of all the physiological, hematological, production and gene expression traits recorded throughout the study period.

Variable	No of Observations	Mean	Std Dev	Minimum	Maximum
PR (beats/min)	69	72.77	10.39	50	90
RR (breaths/min)	69	27.59	6.37	20	44
RT (°C)	69	37.33	0.67	36.1	39.4
Eye IRT (°C)	66	37.16	0.32	36.1	37.9
Forehead IRT (°C)	66	30.15	0.95	27.2	32.1
Back IRT (°C)	66	31.58	1.37	28.7	34.2
Flank IRT (°C)	66	33.84	1.09	30.3	35.6
Fore-shank IRT (°C)	66	27.75	1.83	24.1	31.1
Right-fore quarter IRT (°C)	66	34.53	1.05	30.7	36.5

Left-fore quarter IRT (°C)	66	34.54	1.05	30.8	36.6
Right-hind quarter IRT (°C)	66	34.49	1.05	30.8	36.7
Left-hind quarter IRT (°C)	66	34.51	1.07	30.7	36.6
PLT (x10^3/μl)	67	272.99	137.30	64	758
MPV (fL)	67	5.70	0.58	4.3	7.3
PDWc (%)	67	17.14	0.69	15.8	18.8
PCT (%)	67	0.15	0.07	0.03	0.42
WBC (x10^3/μl)	67	11.65	4.00	4.7	21.5
Lymphocyte (x10^3/μl)	67	5.32	2.10	1.1	9.6
Monocyte (x10^3/μl)	67	1.19	0.49	0.4	2.8
Granulocyte (x10^3/μl)	67	5.14	2.28	1.8	11.3
RBC (x10^6/μl)	67	5.17	0.89	2.66	7.15
HCT (%)	67	23.68	3.44	13.4	32
RDWc (%)	67	15.99	1.82	14	25.6
Milk yield (L/day)	66	11.08	5.55	3	25
Density (kg/m^3)	52	22.56	3.45	14.9	30.1
Lactose (%)	52	3.90	0.44	3.1	5.2
SNF (%)	52	7.10	0.81	5.6	9.5
Protein (%)	52	2.60	0.30	2.1	3.5
Salts (%)	52	0.59	0.08	0.5	0.8
HSF1 (ΔCt)	36	6.77	0.99	4.09	8.67
HSP70 (ΔCt)	36	2.06	1.88	-2.20	4.94
HSP90 (ΔCt)	36	-0.23	1.30	-2.56	3.25
IL18 (ΔCt)	36	4.65	1.53	1.93	7.88
IFNγ (ΔCt)	36	6.59	1.53	3.39	9.25
IFNβ (ΔCt)	36	4.25	1.31	1.22	6.42
TNFα (ΔCt)	36	3.68	1.16	1.82	6.21
GH (ΔCt)	36	11.96	1.50	8.27	15.03
GHR (ΔCt)	36	8.05	2.16	4.21	12.57
IGF-1 (ΔCt)	36	7.75	2.62	2.83	12.19
LEP (ΔCt)	36	4.23	1.89	0.41	8.18

PR: Pulse rate; RR: Respiration rate; RT: Rectal temperature; IRT: Infrared thermography; PLT: Platelet count; MPV: Mean Platelet Volume; PDWc: Platelet Distribution Width; PCT: Plateletcrit; WBC: White Blood Cell, RBC: Red Blood Cell, HCT: Hematocrit; RDWc: Red Cell Distribution Width; *HSF1: Heat Shock Factor-1*; *HSP70: Heat Shock Protein 70*; *IL18*: *Interleukin 18*; *IFNγ*: *Interferon gamma*; *TNFα*: *Tumor necrosis factor alpha*; *GH*: *Growth hormone*; *GHR*: *Growth hormone receptor*; *IGF-1: Insulin-like growth factor-1* and *LEP: Leptin*.

Table S4. Least square means with standard errors for influence of THI and season on leukocyte and erythrocyte indices of dairy cows.

Effects	WBC (x10^3/μl)	Lymphocyte (x10^3/μl)	Monocyte (x10^3/μl)	Granulocyte (x10^3/μl)	RBC (x106/μl)	HCT (%)	RDWc (%)
Overall mean	11.65 ± 4.00	5.33 ± 2.10	1.19 ± 0.49	5.14 ± 2.28	5.17 ± 0.89	23.68 ± 3.44	15.99 ± 1.82

THI_1-7	NS	NS	NS	NS	NS	NS	NS
1 (THI < 75)	11.44 ± 1.20	5.05 ± 0.52	1.15 ± 0.19	5.13 ± 0.91	5.32 ± 0.22	24.24 ± 1.01	15.98 ± 0.53
2 (THI ≥ 75)	13.15 ± 0.91	5.93 ± 0.42	1.32 ± 0.13	5.76 ± 0.60	5.02 ± 0.17	23.56 ± 0.77	15.56 ± 0.41
Season	NS	NS	NS	NS	NS (0.99)	NS (0.87)	NS
Late summer	12.42 ± 0.71	5.86 ± 0.35	1.22 ± 0.10	5.23 ± 0.42	5.17 ± 0.14	23.83 ± 0.60	15.86 ± 0.33
Early monsoon	12.17 ± 1.00	5.13 ± 0.45	1.26 ± 0.15	5.66 ± 0.72	5.17 ± 0.19	23.98 ± 0.84	15.68 ± 0.45
Lactation stage	NS	NS	NS	NS	NS (0.15)	NS (0.11)	NS
Reference range [44]	4.9-12.0	1.6-5.6	0.0-0.8	1.8-6.3	5.1-7.6	22-33	15.5-19.7

WBC: White Blood Cell, RBC: Red Blood Cell, HCT: Hematocrit; RDWc: Red Cell Distribution Width.
